# Immobility-induced hypercalcemia following complex lower limb trauma: A case report

**DOI:** 10.1016/j.jpra.2025.08.030

**Published:** 2025-08-28

**Authors:** Mai Nishijo, Calver Pang, Keith Anderson, Charles Yuen Yung Loh

**Affiliations:** a Department of Plastic Surgery, Cambridge University Hospital NHS Foundation Trust, Cambridge, United Kingdom; b Department of Surgical Biotechnology, Division of Surgery and Interventional Science, Faculty of Medical Sciences, University College London, London, United Kingdom

**Keywords:** Hypercalcemia, Immobilization, Polytrauma, Lower limb fracture, Calcium monitoring, Trauma rehabilitation

## Abstract

Immobilization-induced hypercalcemia is a rare but potentially life-threatening complication in patients with prolonged immobility, particularly in those with severe trauma. We describe a 22-year-old male polytrauma patient with a Gustilo-Anderson grade 3b open tibial-fibular fracture and traumatic brain injury. During prolonged recovery complicated by multiple surgical interventions, he developed moderate hypercalcemia two months post-injury. Common causes such as malignancy and primary hyperparathyroidism were excluded. Hypercalcemia was attributed to prolonged immobilization and successfully managed with intravenous fluids.

This case highlights the need for routine calcium monitoring in trauma patients with prolonged immobilization. Early recognition and management of hypercalcemia are critical to prevent serious complications.

## Introduction

Immobility-induced hypercalcemia is an uncommon clinical entity, often under-recognized in trauma patients. While typically mild and asymptomatic, severe hypercalcemia can result in serious complications including arrhythmias, neurological dysfunction, and even respiratory arrest.^1^^,^^2^ We report a case of a young polytrauma patient who developed moderate symptomatic hypercalcemia during prolonged immobilization following lower limb reconstruction.

## Case presentation

**Figure 1:**
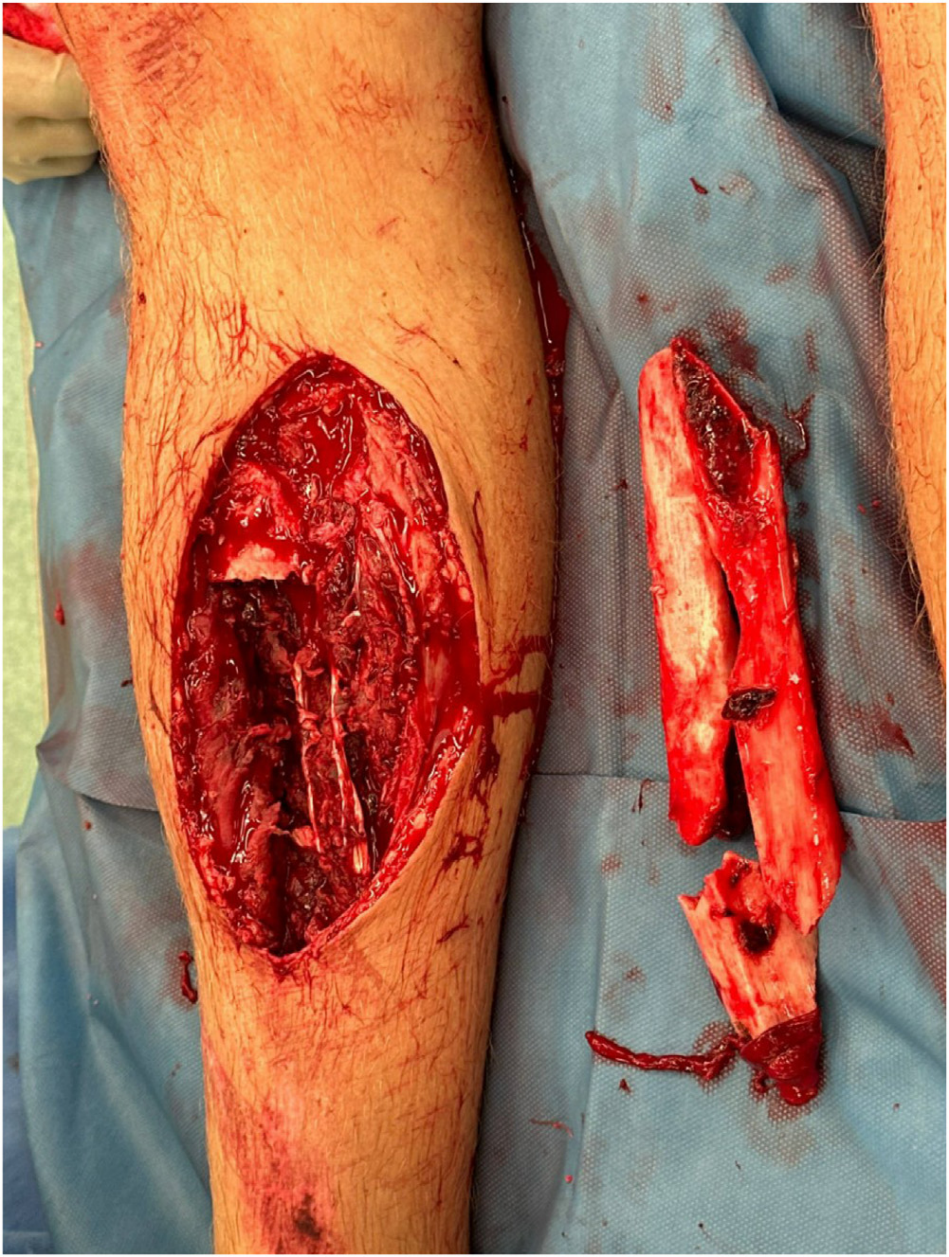
Lower limb open tibial-fibular fracture following initial debridement, demonstrating a 16 cm bone gap.

A 22-year-old male patient presented following a high-energy road traffic accident. Injuries included a Gustilo-Anderson grade 3b open tibial-fibular fracture with significant bone loss, traumatic brain injury (TBI), and multiple closed fractures. Initial orthopedic management involved debridement and application of an external fixator (bone gap 16 cm) (Figure 1), followed by a transposition flap and split-thickness skin graft. He was planned for treatment with a Taylor Spatial Frame and bone transport once the soft tissue defect healed.

Subsequent reconstructive efforts were complicated by soft tissue incarceration, for which a tissue expander was inserted. The expander later extruded, requiring salvage with a free ALT musculocutaneous flap, which unfortunately became congested. Initial management with leech therapy was unsuccessful, necessitating surgical debridement. The subsequent soft tissue defect was managed with a medial gastrocnemius flap and lower limb shortening. The patient is currently undergoing rehabilitation with a plan for bone transport once the soft tissue injury is fully healed.

At two months post-injury, he developed abdominal pain and nausea. Blood tests revealed elevated adjusted serum calcium (2.92 mmol/L, peaking at 3.28 mmol/L). Serial electrocardiography showed no abnormalities. Endocrinology consultation excluded alternative etiologies such as hyperparathyroidism and malignancy. A diagnosis of immobilization-induced hypercalcemia was made. Management consisted of intravenous fluids with gradual normalization of calcium levels over several weeks. Ongoing calcium monitoring was instituted throughout his rehabilitation phase.

## Discussion

This case highlights the importance of regular calcium monitoring in complex trauma patients with prolonged immobility. The exact pathophysiology is not fully understood but is thought to result from increased osteoclastic bone resorption due to the lack of mechanical stress on bone. Furthermore, osteocytes produce elevated levels of sclerostin during inactivity, suppressing osteoblastic bone formation, thereby amplifying calcium release into the circulation.^1^^,^^3^

Although more prevalent in patients with spinal cord injuries, cases have been reported in trauma patients with prolonged bed rest and limited weight-bearing capacity. In this patient, concurrent TBI and reduced physiotherapy participation, along with multiple surgical setbacks, led to significant immobility, likely contributing to the development and severity of hypercalcemia. Given the frequency of prolonged immobilization in complex lower limb reconstructions, plastic and orthopedic surgical teams should be vigilant for this rare but serious metabolic complication.

Management begins with identifying the condition through clinical suspicion and serum calcium measurement. Initial treatment includes intravenous fluids. If ineffective, pharmacological agents such as bisphosphonates, calcitonin, or denosumab may be warranted.^4^^,^^5^ Regular monitoring is essential, particularly during rehabilitation phases, to ensure timely detection and treatment.

## Conclusion

Prolonged immobilization following complex orthopedic trauma can lead to significant hypercalcemia. Clinicians should routinely monitor calcium levels in high-risk trauma patients, as early recognition and prompt management are essential to prevent systemic complications.

## Declaration of competing interest

None declared.
